# Effectiveness of the treatment of depression associated with cancer and neuroimaging changes in depression-related brain regions in patients treated with the mediator-deuterium acupuncture method

**DOI:** 10.1515/biol-2022-0709

**Published:** 2023-11-09

**Authors:** Jianlun Lian, Weiyuan Sun, Fang Dong, Xueliang Zhu, Xue Sun, Songtao Jia, Limin Gao, Meimei Wei

**Affiliations:** Department of Oncology One, Hebei Provincial Hospital of Traditional Chinese Medicine, Shijiazhuang, Hebei, 050011, China; Key Laboratory of Integrated Chinese and Western Medicine for Gastroenterology Research (Hebei), Shijiazhuang, Hebei 050011, China; Ministry of Human Resources, Hebei Provincial Hospital of Traditional Chinese Medicine, Shijiazhuang, Hebei, 050011, China; Department of Brain Disease II, Hebei Provincial Hospital of Traditional Chinese Medicine, Shijiazhuang, Hebei, 050011, China; Functional Department, Hebei Provincial Hospital of Traditional Chinese Medicine, Shijiazhuang, Hebei, 050011, China; Catheter room, Hebei Provincial Hospital of Traditional Chinese Medicine, Shijiazhuang, Hebei, 050011, China; Acupuncture Rehabilitation Department, Hebei Provincial Hospital of Traditional Chinese Medicine, Shijiazhuang, Hebei, 050011, China

**Keywords:** depression, cancer, neuroimaging, brain regions, mediator-deuterium, acupuncture

## Abstract

Cancer patients should be concerned about depression, which can negatively impact their mental health. To develop efficient therapies, it is essential to comprehend the connection between cancer and depression. This study used neuroimaging to investigate the use of mediator-deuterium acupuncture (MDA) for people with cancer-induced depression and its effects on brain regions associated with depression. Resting-state functional magnetic resonance imaging and neurocognitive testing were conducted on the participants, and statistical package for the social sciences was utilized to analyze the behavioral data. Clinical and theoretical data were analyzed to evaluate acupuncture’s effectiveness against gynecological cancer. In the research, there were 40 participants, 20 in each group. Except for psychomotor speed, there was no discernible difference in pre-chemotherapy cognitive test results between patients and healthy controls (HCs). However, there were substantial differences in post-treatment cognition test results, showing that the patient group had progressed. According to longitudinal graph analysis, the patient group’s local and global brain efficiency significantly declined, and lower local efficiency was associated with lower raw Trail Making Test-A results. Furthermore, poorer verbal memory scores were associated with lower overall performance in the sick group but not in the HC group. According to the research, MDA has potential as a supplemental therapy since it may improve brain function and address depression-related neurological abnormalities in cancer patients. More research is required to fully comprehend the variations between cancer and depression-related brain areas during patient therapy, maybe incorporating MDA.

## Graphical abstract

**Figure:**
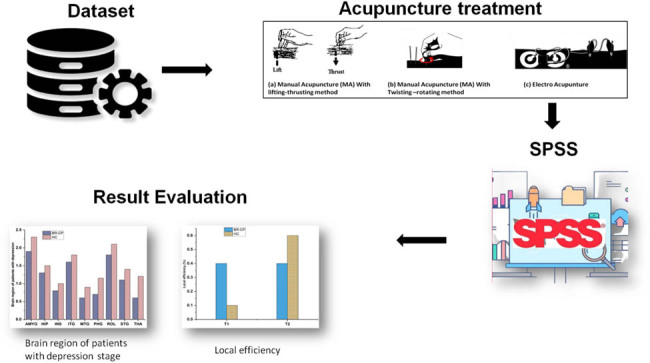

## 1 Introduction

**Figure 1:**
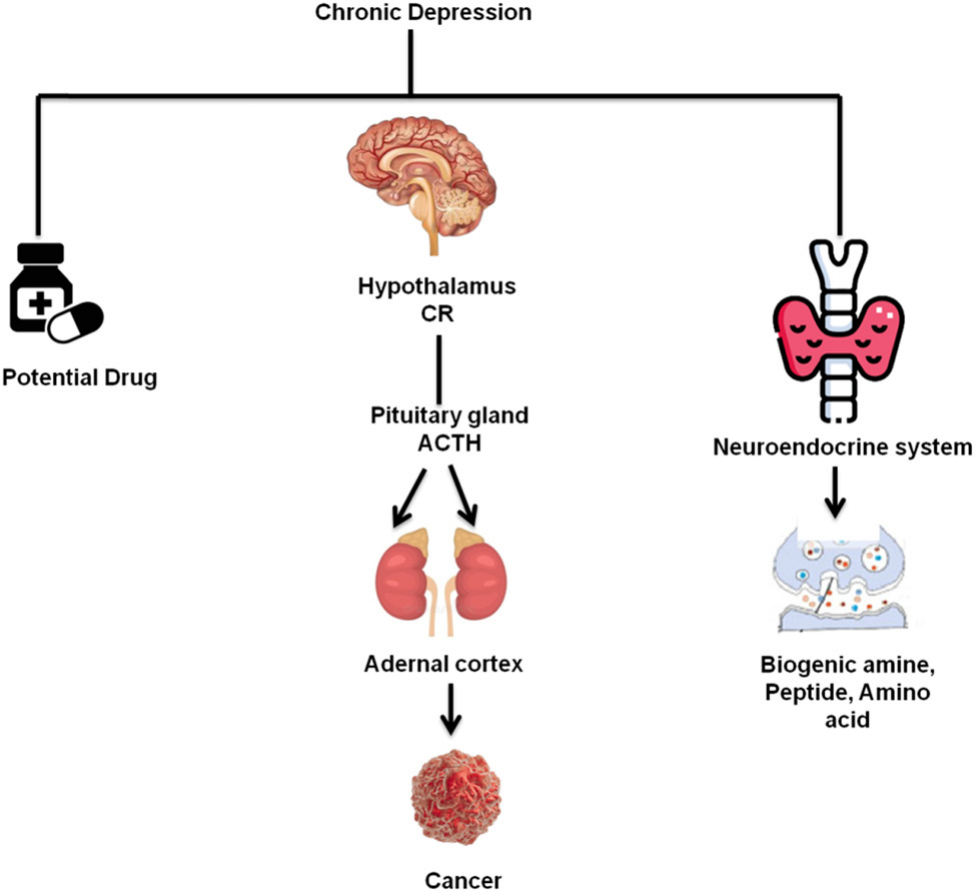
General framework of brain region with cancer.

Many individuals have become either cancer free or have had their condition managed for much longer because of patient survival advances in cancer therapy. Furthermore, if people live longer, there will be more cancer survivors. The morbidity and death rates have increased due to cancer treatment’s short- and long-term negative effects [1]. The term “disease-related malnutrition” refers to a condition that develops when an underlying illness, such as cancer, triggers the body’s inflammatory response. Anorexia and tissue degeneration are brought on by this inflammatory response, which may also change body composition, cause considerable weight loss, and impair functional ability. Cancer cachexia is a multifactorial illness specifically defined by an uncontrollable, long-term loss of weight and skeletal muscle mass, alone or in conjunction with a loss of fat mass. It results in significant functional deterioration and cannot be entirely reversed by traditional dietary treatment. Precachexia, cachexia, and refractory cachexia are the different phases of cancer cachexia. Anorexia and glucose intolerance are two early clinical and metabolic markers of pre-cachexia before decreasing body fat and lean muscle mass. The likelihood of developing cachexia varies and relies on the kind of cancer, the stage of the illness, the level of systemic inflammation, the amount of antineoplastic medication received, and the patient’s reaction to it. Highly advanced or quickly progressing cancer that does not respond to therapy might cause refractory cachexia. At this point, controlling weight loss actively is no longer feasible, and the prognosis is less than 3 months [2]. The symptoms of depression include changes in a person’s thoughts, feelings, and mood and how these things adversely affect their health. Over 300 million individuals suffer from depression globally. This is equivalent to 4.3% of the global population. The Global Health Estimates 2017 report from the WHO estimates that 4.5% of Indians are depressed. One of the most prevalent brain illnesses is this one. It may impact anyone directly, indirectly, or via a buddy [3]. Neuroimaging has become more important in clinical diagnosis and fundamental biological research over the past few decades. The four imaging techniques that are used the most frequently are positron emission tomography (PET), single-photon emission computed tomography, computerized tomography, and magnetic resonance imaging (MRI), as is covered in the section below. Because of MRI, several new imaging modalities have been developed lately, including diffusion-weighted imaging, diffusion tensor imaging, susceptibility-weighted imaging, and spectroscopic imaging, a non-invasive, non-radioactive, and flexible imaging technology. Figure 1 depicts the general framework of brain regions with cancer.

PET is especially flexible since it can use a variety of radiotracers to target different substances or track different physiological pathways used by the body’s receptors [4]. A crucial component of Traditional Chinese medicine is acupuncture. Acupuncture has emerged as one of the most recognizable complementary and alternative therapies since its effectiveness has been acknowledged globally. Most acupuncture prescriptions used in clinical settings include at least two acupoints. Acupoint combinations are more effective overall than single acupoints, according to prior studies. Combinations of acupoints were superior to single acupoints from macroscopic viewpoints, and animal investigations supported these findings at the molecular level. The approach utilized most frequently in the pertinent studies is fMRI. Some researchers using resting-state functional magnetic resonance imaging (Rs-fMRI) claim that the therapeutic effect of combining acupoints is not simply the accumulation of several single acupoints; rather, it has a synergistic effect by affecting more brain regions than the sum of the individual acupoints, and the altered areas differ from the results of single acupoints groups [5].

Before receiving treatment, individuals with metastatic non-nervous system cancer of the non-spinal cord had their topological brain structural network and emotional characteristics evaluated [6]. Depression sufferers exhibit brain morphological abnormalities. Despite the need for therapeutic targets and procedures, the molecular causes of structural abnormalities are unknown. They examined depression-related gray matter volume (GMV) changes and molecular composition [7]. According to the study [8], it is possible to use neural targets and inflammatory and gastrointestinal biomarkers to predict if obese people may experience depression and related anxiety. Analysis [9] used ML algorithms in the neuroimaging areas for depressive disorders and searched PubMed for pertinent publications that addressed depressive disorders and were published up until December 2021. According to the study [10], survivors of childhood sarcoma may have cortical atrophy and diminished functional coherence, but it may be essential for both sustained attentional function and regular emotional pain. During adolescence, the frontal areas may be particularly sensitive. The cortical brain alterations in adult survivors were examined in research [11]. To get structural and functional brain information, they analyzed “high-resolution structural (*T*1-weighted) MRI” and “rsfMRI” among patients who survived childhood sarcoma after receiving “high-dose intravenous chemotherapy.” The study [12] examined brain activity alterations in cancer patients with secondary depression. A study [13] covered the possibility of using stem cells to treat and comprehend the causes of sadness. A person in excruciating pain may not function well in their social, professional, or personal lives. Antidepressants regulate the amounts of “serotonin, norepinephrine, and dopamine” in the brain to treat clinical depression. It has been demonstrated that peripheral inflammation may reflect central inflammation in astrocytes and microglia, causing persistent levels of inflammation in the brain. Recent research [14] has connected neuroinflammatory pathways to the origins of depression. The study [15] aimed to examine how gender affects cancer patients and how depressive symptoms are related to gender-related cancer variables. Antidepressant drugs’ therapeutic effects on the brain are thought to be mostly independent of the structural changes that they cause in the brain [16]. The study looked at long-term neuroimaging studies [17] to better understand the neurobiological mechanisms underlying the clinical emergence of cancer-related cognitive impairment in breast cancer patients. The genetic differences between Alzheimer’s disease (AD) and two age-related cancers, breast and prostate, were examined in the study [18] to identify single-nucleotide polymorphisms that are connected to the inverse comorbidity of AD and cancer. The cognitive profile of “pediatric low-grade glioma” patients treated without radiation therapy was analyzed in the study [19], along with white matter structure, neuropsychological performance, and medical and demographic factors that influence functioning. As a result, we concentrated on the efficacy of treating cancer-related depression and neuroimaging alterations in brain areas linked with depression in patients receiving mediator-deuterium acupuncture (MDA).

## 2 Materials and methods

### 2.1 Data collection

The Unit of Gynecological Oncology recruited all participants at a general teaching hospital. The “third affiliated hospital of Guangzhou Medical University and The Hong Kong Polytechnic University” received approval from their respective ethics committees [20]. Women in this study ranged in age from 18 to 65 from China, and all had been diagnosed with stage I–III gynecological cancer. They were all scheduled to undergo surgery followed by adjuvant chemotherapy.


**Informed consent:** Informed consent has been obtained from all individuals included in this study.
**Ethical approval:** The research related to human use has been complied with all the relevant national regulations, institutional policies and in accordance with the tenets of the Helsinki Declaration, and has been approved by the authors’ institutional review board or equivalent committee.

### 2.2 Criteria for inclusion and exclusion

- Women who had experienced cancer in the past (although not as their first diagnosis), were in the last stages of the disease, or had a significant needle fear were excluded.
- Women under 1 year of age and a menopausal state matching that of the sick group were the inclusion criteria for healthy controls (HCs).
- The use of psychotropic drugs, a history of neurological disease, traumatic brain injury, intellectual disability, or depression or anxiety were all reasons for dismissal from the patient or control groups.

### 2.3 Acquiring MRI data

For neuroimaging studies of cancer and cognition, the International Consortium for Cancer and Cognitive Function (ICCTF) advises using rs-fMRI and high-resolution *T*1-weighted anatomical MRI images in Table 1.

**Table 1:** Information on neuroimaging studies

Neuroimaging study information	
Study type	Cancer and cognition
Consortium	ICCTF
MRI scanner	Philips 3.0 T
Head coil	8-channel SENSE head coil
rs-fMRI data collection	Whole brain
Patient instructions	Close your eyes, take a deep breath, and remain conscious
rs-fMRI method	*T*2-weighted gradient-echo EPI
Number of EPI volumes acquired	240
In-plane imaging resolution	3 mm × 3 mm × 3 mm
In-plane FOV	256 mm × 256 mm
Slice thickness	4 mm
Total axial slices	33
TE (echo time)	30 ms
TR (repetition time)	2,000 ms
Flip angle	90°
Scan duration (rs-fMRI)	8 min and 6 s
*T*1-weighted imaging method	3D fast spoiled-gradient recalled acquisition in steady state
Number of coronal slices acquired	164
TE (echo time)	3.8 ms
TR (repetition time)	8.2 ms
Flip angle	7°
Slice thickness (*T*1-weighted)	1 mm
Slice thickness (thin slices)	1 mm
FOV (field of view)	256 mm × 256 mm
Voxel resolution	1 mm
Acquisition matrix	256 × 256
Scan duration (3D-*T*1)	5 min and 58 s

### 2.4 Acupuncture treatment

By stimulating the body at certain locations, acupuncture works. During treatment, modest electrical stimulation or gentle hand manipulation is used to manipulate the regions of interest after thin steel needles have been placed. These locations may also be heated (moxibustion) or pushed (acupressure). The majority of the body’s systems may benefit from acupuncture, according to contemporary studies. Acupuncture primarily stimulates the neural system, alters how the nervous system interprets pain signals, and releases endorphins and serotonin, endogenous analgesics. Modern scientific researchers have identified the following effects of acupuncture:

- Controlling several physiologic processes;
- Bringing on analgesia;
- Lingual-paralimbic-neocortical network modulation;
- Increasing regional blood flow; and
- Preventing pathogens from entering the body.

As a non-specific treatment, acupuncture has many indications, particularly for functional disorders, contributing to its therapeutic benefits on various systems. The two main forms of acupuncture used to treat cancer are manual MDA and electro-acupuncture (EA), depending on the techniques used for manipulation or stimulation. In manual acupuncture, needles are manually inserted into acupuncture sites (acupoints) and moved to induce a specific needling feeling of pain, numbness, heaviness, and distension called as that is required for acupuncture analgesia and aids patients in receiving the best therapeutic outcomes. Each patient’s requirement and disease severity will determine the frequency and length of MDA treatments. MDA appointments are frequently scheduled regularly, from once or twice weekly to many times per week. Every session could last between half an hour and 1 h. For instance, patients could start getting MDA treatments twice a week for 6 weeks and then have weekly sessions to maintain results. To manage depression linked to cancer and its accompanying neuroimaging abnormalities, each patient’s particular demands and reactions should be considered when medical specialists create the actual treatment plan.

**Figure 2:**
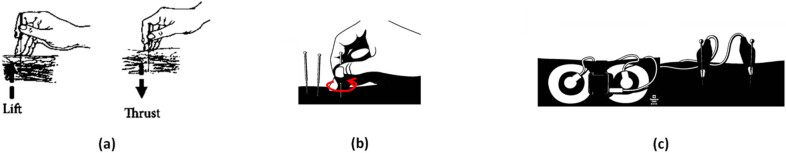
Acupuncture for cancer treatment. (a) Manual acupuncture (MA) with lifting-thrusting method. (b) Manual acupuncture (MA) with twisting–rotating method. (c) Electro acupuncture.

The two manipulation techniques that are most frequently utilized in medical treatment are “lifting-thrusting” (Figure 2a) and “twisting-rotation” (Figure 2b). Different parameters are applied to acupoints using needles attached to an electric stimulus when transdermal electrical stimulation is utilized in EA (Figure 2c), which stimulates nerve fibers and has analgesic effects. The fundamental advantage of EA over MDA is that certain therapeutic benefits, such as analgesic activity, can be achieved by constantly applying predetermined, modifiable parameters (frequency, intensity, and duration). The stimulation of A/A-type afferents is the main element of the peripheral afferent mechanism causing EA analgesia. In the parts of the manual that follow, the precise variations of MDA and the electrical properties of EA will be covered in detail, along with a potential mechanism for how MDA and EA can treat cancer.

### 2.5 Statistical analysis

statistical package for the social sciences (SPSS) for Windows was used for descriptive statistics, correlation analysis, and comparative studies. The descriptive statistics’ means, SDs, and ranges are displayed. Cognitive impairment was present in cancer patients on two or more neurocognitive compared to the HC group; two “tests with a *Z*-score of −1.5” or lower were performed. The *Z*-score was created by multiplying the difference between the raw scores for the subjects and the mean scores for the groups by the standard deviation. The significance of the relationship between brain connectivity and neurocognitive function was assessed using the Pearson correlation coefficients (chi-square test). We analyzed the differences between the groups using independent or paired *t*-tests. Every statistical test was run using a two-tailed test, and outcomes with a *P* value of 0.05 or less were regarded as significant.

#### 2.5.1 Chi-squared test

The Pearson chi-squared test determines if the observed difference in category means is truly coincidental. It examines whether or not the frequency distribution of particular occurrences found in a collection corresponds to an analytical distribution. The options under evaluation must be mutually exclusive and have a one-to-one cumulative probability. It is common for each episode to offer data with classifications, as is the case below. The assumption that a standard dice with six sides is “fair” is, in fact, simplistic. Data consisting of measurements for two components may be considered independent by a contingency board if the board examines independence.

(1) \[{\mathrm{Pearson}}{\mathrm{\mbox{'}}}{\mathrm{s\; chi}}\text{-}{\mathrm{squared\; test}}=\mathop{\sum }\limits_{l=1}^{m}\frac{{({R}_{j}-{H}_{j})}^{2}}{l},]\]

where \[{R}_{j}]\] = metric type values \[j,{R}]\] = metric total, \[{H}_{j}]\] = expected type frequency \[j,{\mathrm{and}}{m}]\] = number of cells.

Pearson then substitutes \[e]\] for \[u]\] in the definition of \[{u}^{2}]\] instead. The multinomial standard deviations and correlation coefficients are covered as follows:

(2) \[{u}^{2}=R\left(\frac{{e}^{2}}{m}\right).]\]

If not, they would have received care. The following is how expected chi-square values are determined:

(3) \[H=\frac{{m}_{{\mathrm{c}}}\times {m}_{{\mathrm{v}}}}{i},]\]

Here, \[H]\] = represents the worth of the unit’s effort, \[{m}_{{\mathrm{c}}}]\] = the cell nucleus row edge is indicated. \[{m}_{{\mathrm{v}}}]\] = indicates the row edge of that cell, and \[i]\] = reflects the sample’s entire population.

The marginal sum for the row and column of each cell separates the number of pieces count.

(4) \[{u}^{2}=\frac{{(N-H)}^{2}}{H},]\]

Correlation metrics are statistical assessments of a link’s strength. The most popular method for determining Chi-square strength is Cramer’s *F* test. It is straightforward to compute using the following formula:

(5) \[\sqrt{\frac{{y}^{2}/m}{(j\left-1)}}=\sqrt{\frac{{y}^{2}}{m(j\left-1)}}.]\]

## 3 Result

A total of 18 potentially qualified gynecological cancer patients were contacted; 15 of them agreed to participate. The requests of the three patients were turned down on the grounds that the neurocognition tests and MRI scans would be too taxing. Fifteen HCs were menopausal and of similar age. With a mean age of 49.6, the HCs were aged 29–59. The evaluation was conducted with all of the HCs present. Table 2 provides comprehensive details on the characteristics of all study participants. Moreover, half of the patients (*n* = 8, 53.3%) in the patient group had been given a cervical cancer diagnosis. As an additional cancer treatment, chemotherapy was administered to all patients.

**Table 2:** Characteristics of all patients

Factors	HC	Cancer patients
Age, years mean ± SD (range)	49.60 ± 8.27	49.33 ± 9.14
**Kind of therapy**		
Surgery + chemotherapy + radiation	—	2
Surgery + chemotherapy	—	13
**Kind of cancers**		
Uterine cancer	—	6
Cervical cancer	—	8
Ovarian cancer	—	1
**Employment status**		
Employed	15	2
Unemployed	—	13
**Menopausal status**		
Premenopausal	8	8
Perimenopausal	1	1
Postmenopausal	6	6
**Excellent education**		
High school university and above	1	1
Primary school or below	—	12
High school	14	2
**Illness level**		
Advanced stage (stage IIIb)	—	3
Middle stage (stage IIb–IIIa)	—	3
Early stage (stage I–IIa)	—	9
**Marital status**		
Never married	1	1
Married	13	14
Divorced	1	—

**Figure 3:**
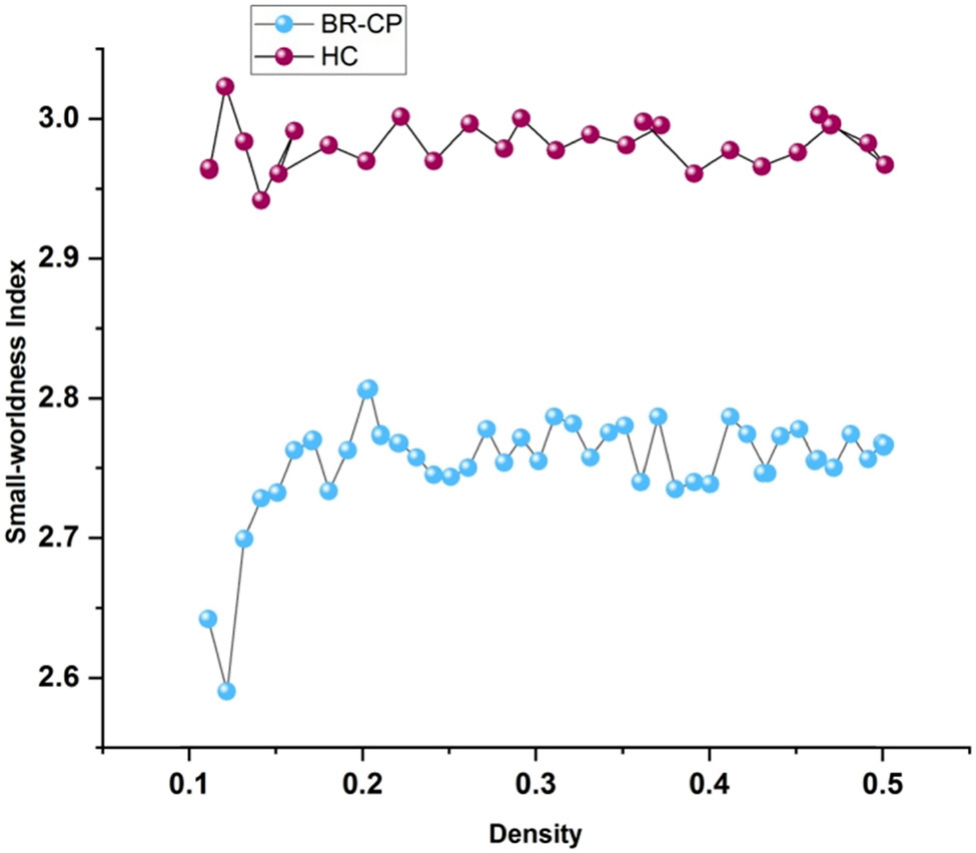
Worldwide connectome integrity.

The small-worldness score for both groups of participants was higher than 1, as shown in Figure 3. Humphries and Gurney claim that small-worldness scores greater than 1 across network densities indicate the presence of small-world connectome organization. In contrast to the HCs, the sick group showed a lower small-worldness measure (Figure 3). In this study, we used features of regional connectomes to determine the average degree of structural connectivity at individual nodes. The Automated Anatomical Labeling atlas template also revealed that across 90 distinct brain locations, the sick group had lower mean node degrees than the HC group.

**Figure 4:**
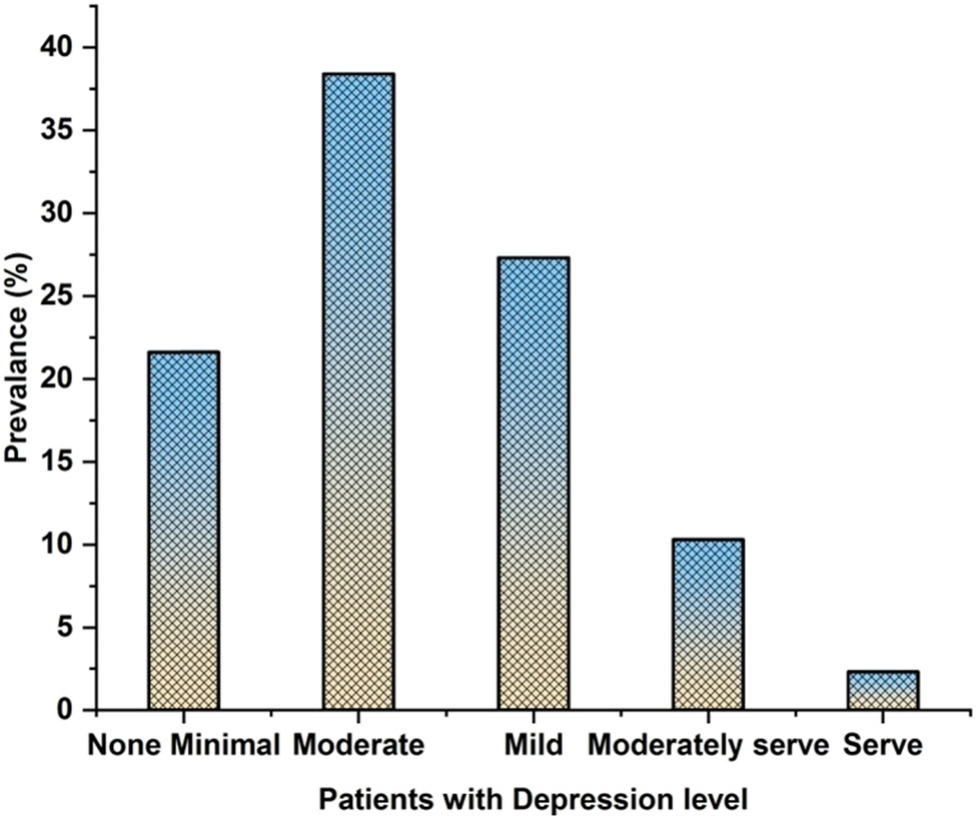
Prevalence of patients with depression level.

Figure 4 depicts the prevalence of patients with depression levels. There 39.2% of people reported having depression or substantial clinical depression. However, additional analyses focused on non-minimal depression, which was present in 21.6% of the population. Numerous demographic and lifestyle factors and comorbid conditions, including sex, occupation, hypertension, a stressful past or present, past or present depressive symptoms, frequent exercise, and loneliness, were linked to non-minimal depression, but depression was unaffected by racial background, degree of education, religion, or diabetes.

**Figure 5:**
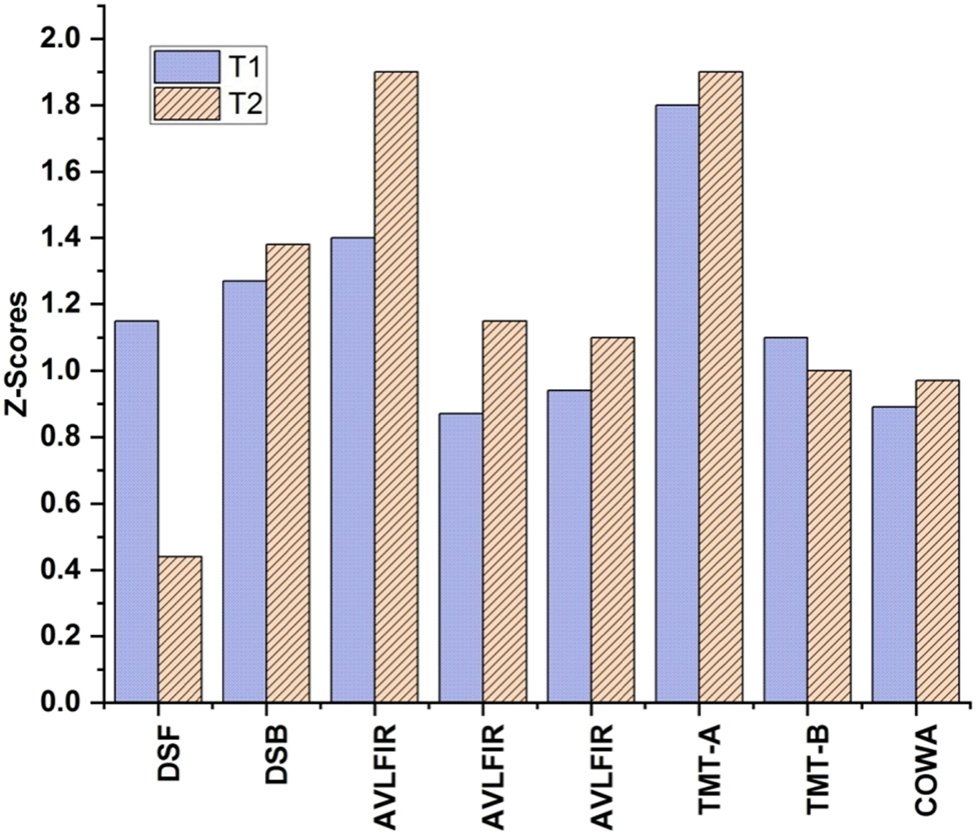
*Z*-scores for all patients.

Table 3 shows the BR-CP and HC tests for cognition average scores at *T*1 and *T*2, except for information processing speed. Neurocognitive assessments showed a significant change at *T*2 (post-chemotherapy) across the board, including on the Digit Span, AVLT, and Trail Making Test (TMT)-A (Figure 5).

**Table 3:** BR-CP and HC tests for cognition average scores at *T*1 and *T*2

Factors	*T*1-mean		*P*-value	*T*2-mean		*P*-value
	HC	BR-CP		HC	BR-CP	
**Language**						
COWA	32.56	33.65	0.32	26.66	16.56	0.37
**Verbal memory**						
AVLT immediate recall	5.62	5.43	0.44	8.16	6.11	<0.02
AVLT delayed recall	5.56	5.96	0.53	8.36	5.02	0.02
AVLT recognition	11.41	11.36	0.93	11.41	8.56	0.32
**Executive function**						
TMT-B	58.81	72.04	0.08	57.73	74.34	0.12
**Attention and working memory**						
Digit span forward	7.30	6.75	0.45	7.53	6.65	0.03
Digit backward	3.15	2.45	0.26	4.26	2.40	<0.02
**Information processing speed**						
TMT-A	43.94	56.64	0.04	39.84	55.21	0.05

The raw patient scores were transformed into *Z*-scores by dividing the standard deviation and then subtracting from the *T*1 mean of the control group. *T*1 and *T*2, cognitive test *Z*-scores, adjusted for occupation and level of education.

Table 4 demonstrates a statistically significant difference in *Z*-scores for cognition tests at *T*1 and *T*2 between patients and HCs. In Table 5, there were cognitive issues in seven individuals at *T*1 and nine patients at *T*2.

**Table 4:** Cancer patients’ *T*1–*T*2 depression test *Z-*scores

Factors	Patients at *T*1	Patients at *T*2	*P*-values
**Executive function**			
TMT-B	−0.18	−0.16	0.19
**Language**			
COWA	0.03	−0.05	0.14
**Verbal memory**			
AVLT recognition	0.43	−0.53	<0.02
AVLT immediate recall	0.82	−0.12	<0.02
AVLT delayed recall	0.73	−0.47	<0.02
**Attention and working memory**			
Digit span forward	−0.23	−0.33	0.03
Digit backward	−0.24	− 0.07	0.02
**Information processing speed**			
TMT-A	−0.15	−0.21	0.04

**Table 5:** *T*1 and *T*2 degeneration of cognition rate

Brain areas	Brain malfunctions	
	*T*2	*T*1
Verbal fluency	2	2
Verbal and learning memory	4	3
Information processing speed	2	2
Attention and working memory	4	3
Executive function	2	2

The small-world organization was present in all patients and HCs, as demonstrated by values of small-worldness greater than 1. Between BR-CP and HC, noticeable variations in “small-worldness” at periods *T*1 and *T*2, as seen in Table 5; between patients and wholesome controls, the typical route length at *T*2 increased significantly (*P* = 0.01). The patient group’s local and global efficiency was decreasing due to the longitudinal graph analysis in Table 6.

**Table 6:** Cognitive global measurements of BR-CP and HC at *T*1 and *T*2

Methods	*T*1		*P*-value	*T*2		*P*-value
	HC	BR-CP		HC	BR-CP	
Local efficiency	0.1	0.38	0.27	0.38	0.58	<0.02
Global efficiency	0.38	0.6	0.65	0.28	0.38	0.46
Small-worldness	1.88	1.64	0.05	1.85	1.56	0.03
Characteristic path length	1.13	0.97	0.29	0.97	1.33	0.02

The correlation between the sick group’s lower verbal memory scores and overall performance was statistically significant, but it was not present in the HC group. Low raw TMT-A scores and poor local efficiency correlated statistically significantly.

**Figure 6:**
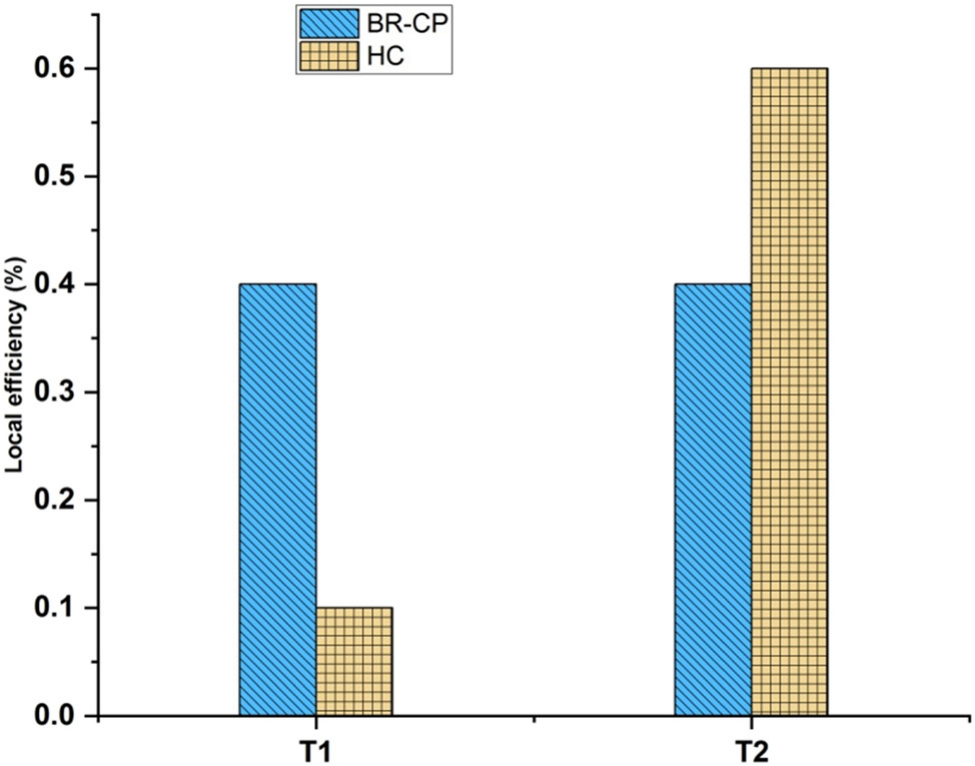
Local efficiency.

Figure 6 depicts the local efficiency. Thus, BR-CP achieved 0.38, HC earned 0.1 for *T*1, BR-CP achieved 0.38, and HC achieved 0.58 for *T*2. Local efficiency measures the small-scale resilience of a network. That is a node’s local efficiency. Describes how well its neighbors can communicate information when it is eliminated. A scaled indicator of local efficiency, ranging from 0 to 1, indicates the network’s highest local efficiency. High local efficiency in functional brain networks reveals a topological structure suggestive of segregated neural processing. It was discovered that the indicated brain region for cancer patients (BR-CP) is compared to the HC in use, such as *T*1 and *T*2. The stated *T*1 is higher than the estimated *T*2.

**Figure 7:**
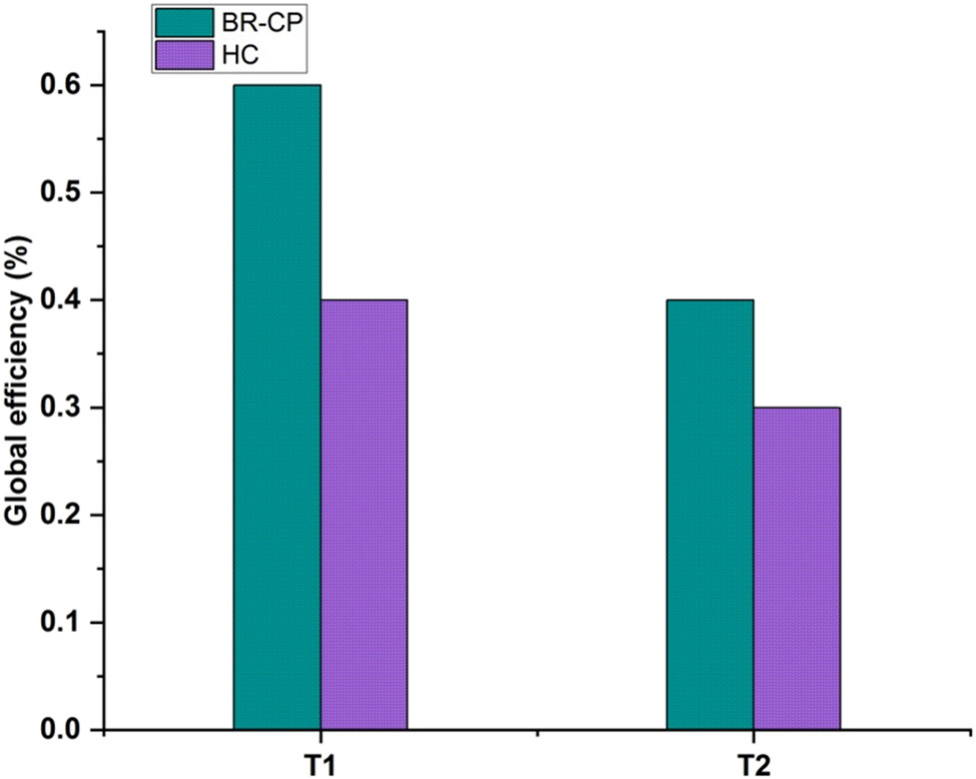
Global efficiency.

Figure 7 depicts global efficiency. Thus, BR-CP achieved 0.6, HC achieved 0.38 for *T*1, BR-CP achieved 0.38, and HC achieved 0.28 for *T*2. According to the efficiency principle, an activity will provide the greatest benefit when the marginal social costs and benefits of its resource allocation are equal. Global efficiency has been used to improve brain connections and transportation systems. The global efficiency, inverse to the characteristic route length, is the network’s average inverse shortest path length. It was discovered that the indicated BR-CP is compared to the HC in use, such as *T*1 and *T*2. The stated *T*1 is higher than the estimated *T*2.

**Figure 8:**
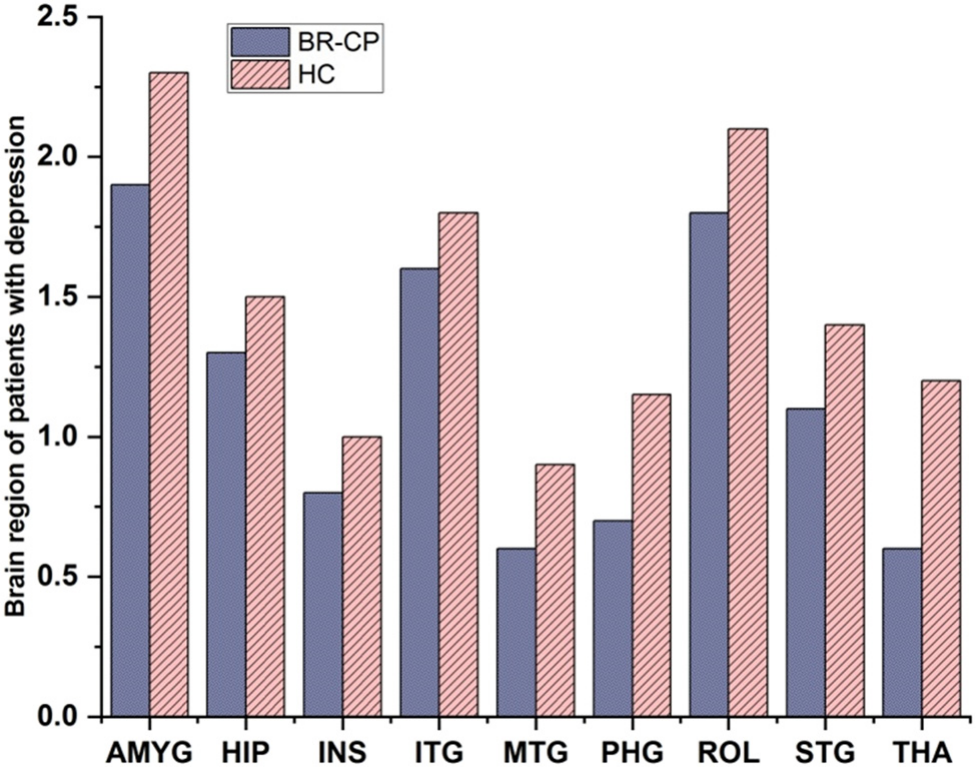
Brain region of patients with depression stage.

Figure 8 depicts the brain region of patients with depression stage. The amygdala, a brain region critical for regulating emotional responses, is the primary source of activating input. Therefore, the HPA axis, which consists of the “hypothalamus, pituitary gland, and adrenal cortex,” may get activated when a person is experiencing emotional distress; to start the stress response. There is growing research that suggests depression shrinks certain brain regions. GMV specifically decreases in these areas. There are many neurons in the brain. GMV loss appears to be more common in people whose depressive symptoms are severe and persistent. As shown in Figure 2, the temporal areas of the brain serve as the functional hubs for cancer patients, while the parietal regions serve as the operational hubs for HCs.

**Figure 9:**
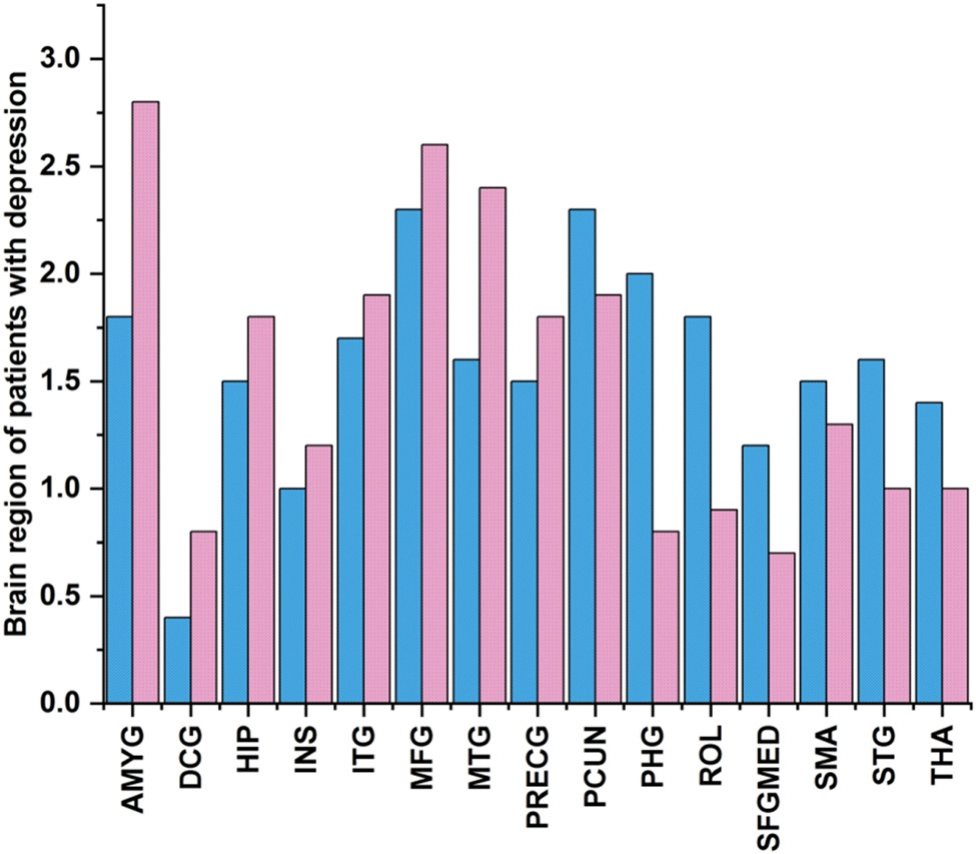
Neurocognitive impairment connecting brain areas.

Areas of the brain were classified as hubs in the networks of the study participants if their node degree values were greater than 1.5 deviations from the mean in a network. Figure 9 shows the hub features of various brain areas. In this patient population, those with neurocognitive impairment tend to show hyperactivity in the “left hippocampus, left parahippocampal gyrus, left and right insula, middle temporal gyrus, and superior temporal gyrus.”

**Figure 10:**
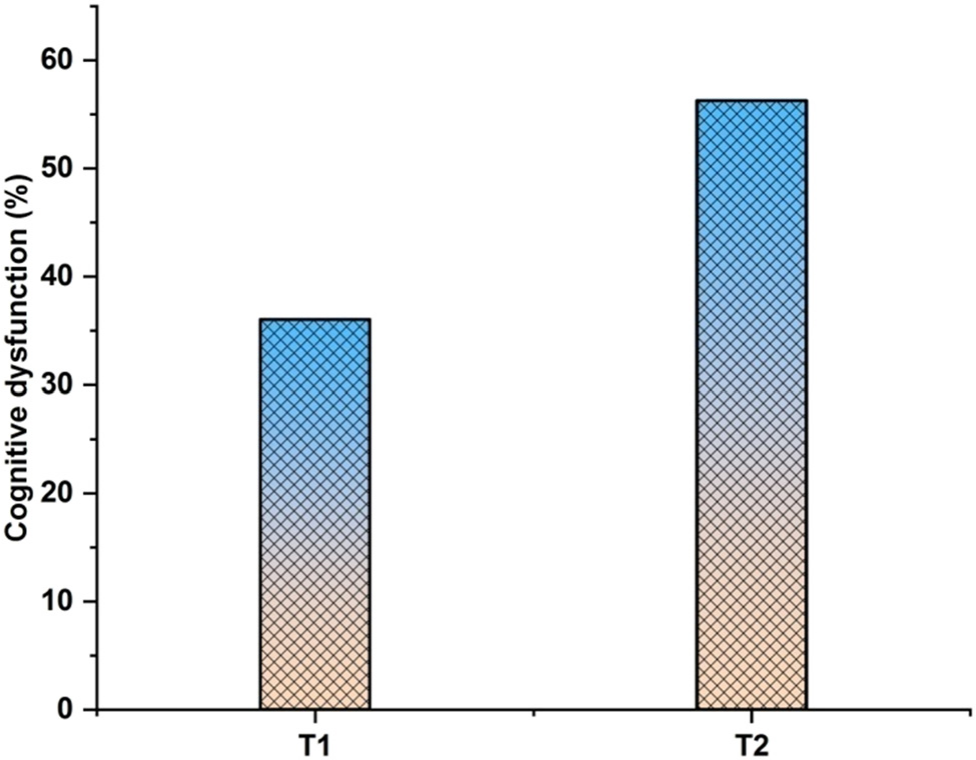
Cognitive dysfunction.

Figure 10 depicts cognitive dysfunction. Cognitive dysfunction affects a person’s ability to pay attention, learn verbally and nonverbally, store information in short-term and working memory, process visual and auditory information, solve problems quickly, and move their body. A range of mental health conditions known as cognitive disorders or neurocognitive disorders affect learning, memory, perception, and problem-solving. It was discovered that the indicated brain area for cancer patients is compared to the previously used techniques, such as *T*1 (0.38) and *T*2 (0.57). The stated *T*2 is higher than the estimated *T*1.

## 4 Discussion

This is the first study to examine the brain functional networks in Chinese women with gynecological cancer while including an HC group with comparable demographics, repeating rs-fMRI assessments, and using a longitudinal graph theoretical approach. According to this study’s findings [21], gynecological cancer patients performed worse on cognition tests after chemotherapy than age-matched, HCs. These findings were consistent with other research on cancer patients after chemotherapy. However, this study’s patient group’s mean score changes on cognitive tests were minimal. Given that all of the patients at the baseline had surgery, it may be caused by cognitive impairment associated with surgery. According to another study [22], individuals with cancer who have local surgery experience more cognitive impairment than at baseline. Patients with gynecological cancer, in particular, showed altered small-world features. Information processing that is both locally specialized and globally interconnected benefits from functional networks with observable small-world characteristics. A substantial correlation between lower raw TMT-A scores and lower local network efficiency indicates that a higher small-worldness index has been associated with slower processing speeds in cancer patients. Both cancer patients and controls demonstrated small-world features of functional brain networks, while local efficiencies of cancer patients were noticeably higher after chemotherapy. Enhancing local performance in cancer patients may impede the flow of information across distant brain areas, as there is a correlation between decreasing network attack tolerance and increased neurocognitive impairment in these patients. The sick group was the only one in which this investigation discovered considerably lower global efficiency and statistically positive associations between lower global efficiency and worse memory scores. The study’s results aligned with earlier research, which showed that chemotherapy-treated breast cancer survivors had less efficient functional brain networks than HCs in response to a neurodegenerative simulation. This study [23] found that the frontal and parietal lobes of healthy people and the temporal lobes of people with mental illness play crucial roles in cognition. This study’s findings allowed researchers to identify functional hubs for individuals with and without cognitive impairments and to distinguish between patients’ networks with and without corners. Patients with cognitive dysfunction have been found to have hyperactivity in the “parahippocampal gyrus, middle temporal gyrus, superior temporal gyrus, and left hippocampus.” These brain regions are crucial for network resilience and information flow regulation because they serve as bridges between various networks. The most vulnerable elements of local functional networks were found to be hubs or highly connected areas of the brain. When taken as a whole, these findings suggest that each of these brain hubs plays a crucial role in the pathophysiology of cognitive impairment and that these regions’ connectome characteristics may, in certain cases, be used to predict neurocognitive performance. The results of this investigation provide fresh information about the causes of cognitive decline in cancer patients [24]. It was necessary to evaluate the significance of brain neuroimaging data about neurocognitive performance to identify the patterns of brain functional networks implicated in neurocognitive impairment. Using Rs-fMRI to predict who may suffer permanent brain damage due to cancer treatment appears promising. Measures of the connectome that result from rs-fMRI have high levels of consistency between tests. In addition, the rs-fMRI scan could be acquired in about 8 min, making it practical for use in busy clinical settings. Neurocognitive outcomes and functional network connectome characteristics have been shown to vary over time in response to therapy [25], and rs-fMRI has been proposed as a tool for studying these changes longitudinally. The study determined that MDA was effective in treating cancer-induced depression, and this conclusion was corroborated by neuroimaging results that demonstrated alterations in brain areas associated with depression. Compared to HCs, neurocognitive testing following MDA showed improved verbal memory and psychomotor speed. A longitudinal graph analysis showed improved brain function after therapy. The findings also pointed to MDA as a possible helpful treatment for cancer patients by indicating a link between changed brain chemistry and depression-related alterations.

## 5 Conclusion

The first long-term analysis into the relationship between gynecological cancer and alterations in brain functional networks and neurocognitive functions in Chinese females is presented in this study. Each participant had a neurocognition assessment and an Rs-fMRI scan. SPSS was utilized for comparison, correlation, and descriptive statistics on behavioral data. We analyzed clinical and basic evidence to comprehensively understand acupuncture’s effectiveness against gynecological cancer. Finally, we reviewed the neurotransmitters, receptors, enzymes, and substances involved in the regional and neurological anticancer effects of MDA. The findings of this study indicate that educating patients about the possibility that chemotherapy may impair their brain function and neurocognitive abilities may help them choose a course of treatment. It may also help select a cohort that would be an appropriate participant for an experimental trial. The study also makes a distinctive contribution by using a multimodality imaging technique to provide light on neurological cancer and/or its treatment are the root cause of cognitive impairment in humans. The larger number of samples would increase the generalizability and statistical power. The lack of a placebo control group might increase biases, making it more difficult to attribute outcomes solely to MDA. The findings of this research may help inform the design of future rehabilitation and treatment programs for this at-risk group.
